# Antibacterial cellulose composite: using SET-LRP for cellulose surface modification with quaternized poly-dimethylammoniumethylacrylate

**DOI:** 10.1039/d6ra03699f

**Published:** 2026-07-08

**Authors:** Enguerrand Barba, J. Benedikt Mietner, Dhanya Raveendran, Benedikt Sochor, Sarathlal Koyiloth Vayalil, Calvin Tu, Christel Vollstedt, Wolfgang Streit, Stephan V. Roth, Julien R. G. Navarro

**Affiliations:** a Institute of Wood Science, Universität Hamburg Hamburg Germany julien.navarro@uni-hamburg.de; b Deutsches Elektronen-Synchrotron DESY Notkestrasse 85 22607 Hamburg Germany; c Advanced Light Source, Lawrence Berkeley National Laboratory 6 Cyclotron Rd Berkeley CA 94720 USA; d Applied Science Cluster, UPES Dehradun Uttarakhand 248007 India; e Institute of Plant Sciences and Microbiology, Universität Hamburg Hamburg Germany; f KTH Royal Institute of Technology, Department of Fibre and Polymer Technology Teknikringen 56-58 10044 Stockholm Sweden

## Abstract

The aim of this study is to graft quaternized poly-dimethylammoniumethylacrylate (PDMAEAQ) onto the surface of cellulose in order to both increase its compatibility with a PVC matrix and provide it with antibacterial properties. Cellulose was first prepared by grafting 2-bromoisobutyric acid to its surface to make it into an initiator for Single Electron Transfer Living Radical Polymerization (SET-LRP), which was then performed using dimethylaminoethylacrylate (DMAEA) as the monomer. The DMAEA units were then quaternized using 1-bromododecane. The resulting product was finally compounded with PVC. The antibacterial properties of the composites were studied, showing antibacterial activity against several bacterial test strains. Then, the modified cellulose/matrix compatibility and basic information on the composite were obtained using SAXS, and water contact angle measurements were used to explain the increased compatibility. Finally, the 3D printability of both PVC and the modified cellulose/PVC composite was tested, showing that, while PVC poses significant challenges for printing, a composite using another matrix would have great potential for everyday applications.

## Abstract 2

Quaternized poly-dimethylammoniumethylacrylate was grafted on the surface of cellulose fibres through SET-LRP to produce an antibacterial composite with PVC. The success of the grafting, antibacterial properties and printability were then assessed.

## Graphical abstract

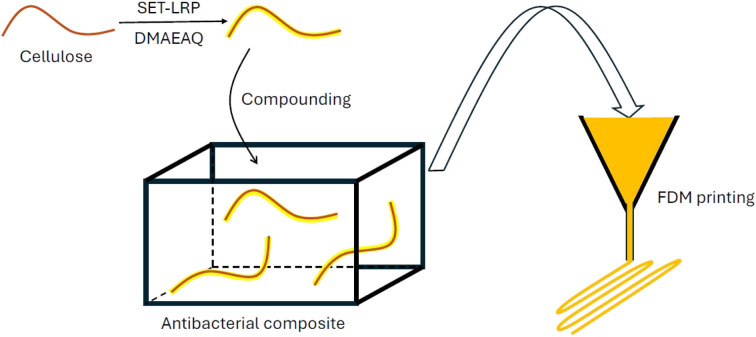

## 1. Introduction

Additive manufacturing technology (also known as 3D printing) was first developed in the early 1980s by Hideo Kodama and has shown great development since then. In 2010, when the original patents fell into the public domain, accessibility and innovations in 3D printing have skyrocketed, together with an increase in printing quality and a decrease in price.^1,2^ The 3D printing technology can now be used in many fields, such as the recycling of used plastics *via* reextrusion,^3^ small batches of products, or on-demand products like clothing,^4^ as well as rapid prototyping (replacement parts, shoes).^5,6^ In addition, its industrial applications are widespread in the building,^7^ automotive,^8^ and aeronautic^9–11^ and defense sectors.^12^ Finally, as the price of printers decreased and became affordable for the general public, 3D printing has become a hobby^13^ for the printing of decorative items or small practical items. This has reached a point where even some main companies in phone manufacturing have taken it into account, such as Nokia releasing a phone case model to 3D print in 2012,^14^ or Samsung funding 3D printing teaching in Europe.^15^ Nowadays, thousands of phone accessory models for printing can easily be found on the internet. One of the main methods for printing plastics is the Fused Deposition Modeling (FDM) technique. It consists of melting a thermoplastic polymer filament through a heated nozzle in order to print a layer of material. By printing the thermoplastic filament layer-by-layer, a three-dimensional structure is created. By changing the layer thickness, the resolution of the print can be controlled. The choice of the polymer used for this printing is crucial for determining its printability and the final properties of the printed object.

There is a great interest in material chemistry for the design of Wood Plastic Composites (WPCs).^16,17^ WPCs are obtained through the composition of wood particles and a plastic, most commonly, a thermoplastic matrix. Cellulose flour and cellulose fibers are easily accessible, biodegradable, renewable, and inexpensive. Because of their good mechanical properties,^18^ cellulose fibers are a good candidate to reinforce polymers. However, because of its hydrophilic nature, cellulose has poor compatibility with most thermoplastic polymers, which are hydrophobic. Several chemical pathways have been developed to transform the hydroxyl groups of the cellulose into more hydrophobic functions^22^ and solve the wood-filler-to-matrix compatibility problem. The two most common ways to do so are the direct reaction of the hydroxyl group to produce another chemical function (silane coupling,^19^ peroxide modification,^20^ acetylation,^19^*etc.*)^21^ or the use of a coupling agent (polymer-grafted maleic anhydride,^22^ methylenediphenyl 4,4′-diisocyanate,^23^*etc.*). Despite the ease of use of these reactions, the introduction of new functionalities onto the surface of the cellulose fiber remains limited. Those reactions will mainly increase the polymer–fiber compatibility. For this reason, finding a new pathway to modify the cellulose fibers with polymers to (A) increase the polymer–fiber adhesion and (B) graft new functional groups to introduce new properties is essential.

Single Electron Transfer Living Radical Polymerization (SET-LRP) is a reaction that has been gaining increasing interest over the past few decades.^24^ It has many advantages, such as its polyvalence (it can be applied to many classes of monomer^25–30^) and its robustness (it works in many reaction media, such as a wide variety of polar solvents, like DMSO,^26^ water,^31^ and even many untypical solvents, like various alcoholic drinks,^32^ blood serum,^33^ and ionic liquids^34^). Thanks to it being a “living” radical polymerization, it is possible to have great control over the final polymer product through the number and addition order of the used monomer(s), ranging from homo-polymers to statistical and block co-polymers. Finally, since the catalyst is added as Cu(0), it can be introduced in solid form as a copper wire, which greatly simplifies the purification step.

Regarding the introduction of antibacterial properties to 3D-printed objects, several methods have been reported. Among them are the loading of the matrix with silver in the shape of nanoparticles,^35–37^ phosphates^38^ or organic molecules with antibacterial properties.^39,40^ However, some of these loading methods, while extremely efficient, have decreasing efficiency with time or exposed toxicity due to the release of antibacterial agents during their activity.^35^ Another method of matrix loading is by oil coating of its pellets before extrusion into a printable filament.^41^ Finally, a common pathway is the surface modification of the printed object with an antibacterial layer, such as ε-poly-l-lysine (EPL) and antimicrobial polypeptide^42^ or a mixture of polyvinylpyrrolidone (PVP) and penicillin.^43^ Among the many antibacterial products, quaternized dimethyl ammonium ethyl acrylate (DMAEAQ) was chosen because of its added antibacterial properties after quaternization.^44,45^ DMAEA is compatible with SET-LRP and can therefore be polymerized on the surface of cellulose fibers.

The aim of this paper is to describe the grafting of quaternized poly-DMAEA (PDMAEAQ) on the surface of cellulose flour and cellulose fiber, followed by the compounding of this modified cellulose with PVC. The composite will then be studied to assess its antibacterial properties and 3D printability, as well as its modified fiber–matrix compatibility.

PVC was chosen as a matrix because of its wide range of applications. While the majority of them are not compatible with 3D printing (pipes, cabling, and construction), its use for packaging would benefit from additive manufacturing and antibacterial properties. The strength of this method is its adaptability and modularity. Additionally, despite it not being the focus of this paper, SET-LRP has been shown to not only be compatible with a wide range of monomers, but also to be easily usable to build block polymers,^46,47^ which would allow for better tuning of the fiber's properties.

A bromine initiator was first grafted onto the surface of the cellulose to serve as a starting point for the SET-LRP of the DMAEA monomer. The product from this SET-LRP (CF-PDAMAEA) was then quaternized using iodododecane, and this final product was compounded in PVC. All synthesis steps were followed by FTIR and solid-state NMR. Finally, the antibacterial properties, printability, and fiber–matrix compatibility of the composite were studied and compared to neat PVC.

## 2. Experimental section

### 2.1. Materials

DMSO at 99%, *N*,*N*-dimethylformamide (DMF) at 99%, imidazole (99%), 2-bromoisobutyric acid (98%), 2-(dimethylamino)ethyl acrylate (98%) stab. with *ca.* 0,1% 4-methoxy-phenol, 1-bromododecane (98%), and copper wire (1.0 mm diameter, annealed, 99.9% (metal basis)) were purchased from Alfa Aesar. 2-Propanol at 99.5% was purchased from J.T. Baker. The cellulose flour CW 630 PU/ARBOCEL was thankfully donated by JRS Rettenmaier. The cellulose fibers were produced using ECF (elemental chlorine-free) bleached kraft softwood pulp from MERCER Stendal GmdH. Poly(vinyl chloride) (PVC), low molecular weight, CDI (reagent grade), and octadecyl acrylate (containing 200 ppm monomethyl ether hydroquinone as inhibitor, 97%) were procured from Sigma-Aldrich. Processing agents used for PVC extrusion were Mark CZ2000 from Galata Chemicals, Paraloid K125 ER from Dow Chemical, Loxiol G60 and Loxiol G21 from Emery Oleochemicals, Ligalub GT from Peter Greven GmdH and Licocene PE4201 from Clariant.

### 2.2. Method

#### Production of cellulose flour and cellulose fiber

Cellulose flour was washed in DMSO and then separated by centrifugation (6000 rpm (4427 g), 20 min). This process was repeated until the supernatant was clear.

Cellulose fibers were obtained from the milling (1500 rpm, 5 min) of 10 g of a cellulose sheet with 200 mL of water in a Retsch RS200 grinding machine. The solvent was then exchanged to DMSO by slowly adding DMSO to the cellulose fiber in water until a 1 : 1 water/DMSO ratio was reached, followed by centrifugation. The cellulose was then redispersed in DMSO and centrifuged again, twice. During synthesis and analysis, cellulose flour (CF_flour_) was used, except for SAXS measurements, where the difference between cellulose flour (CF_flour_) and cellulose fibers (CF_fiber_) was thought to be significant. Synthesis was performed the same way whether fibers or flour was used.

#### Synthesis of the CF-based macroinitiator (CF-MI)

The macroinitiator was synthesized according to our previous paper:^48^ 0.1 g of cellulose was dispersed in 7 mL of DMSO. The solution was then degassed with N_2_, heated at 55 °C, and 3 g of imidazole were added to it. Simultaneously, 4 g of 2-bromo-isobutyric acid was dissolved in 20 mL of DMSO. This solution was also degassed with N_2_, and then 4 g of CDI was slowly added to it. It was then left for 30 minutes to 1 hour, until the gas emission stopped. The 2-bromo-isobutyric acid solution was then slowly poured into the cellulose solution. The resulting solution was degassed with N_2_, then left to react overnight under stirring. Once the reaction was finished, it was centrifuged at 6000 rpm (4427 g) for 20 minutes and then washed by 8 cycles of dispersion in DMSO followed by precipitation by centrifugation (20 minutes, 6000 rpm (4427 g)) and supernatant removal.

#### Procedure for SET-LRP grafting of DMAEA onto CF (CF-DMAEA)

First, 2.34 mL of DMAEA was dissolved in 15 mL of toluene and passed through a column of basic aluminum oxide to remove the stabilizing agent. The column was then washed with 15 mL of solvent to recover as much monomer as possible. A 6 cm long copper wire was coiled in a spring-like shape. It was immersed in conc. HCl for 10 min, washed with water, and then immersed in acetone. Finally, it was dried by air-blowing and used as such. Then, 5 mL of DMSO was degassed using N_2_ and 100 µL of Me_6_-TREN was added under Schlenk conditions. Following this, 0.1 g of cellulose macro-initiator was dispersed in 15 mL of DMSO. The copper wire and the monomer were added to the solution, which was then heated to 40 °C and degassed with N_2_. Once oxygen had been removed from the solution, 0.2 mL of ligand solution was added, and the solution was left to react overnight under stirring. Once the reaction was over, the solid product was recovered by removal of the liquid phase. It was dissolved in toluene and precipitated with isopropanol. It was recovered by centrifugation (20 minutes; 6000 rpm (4427 g)) and then centrifuged (20 minutes; 6000 rpm (4427 g)) twice in isopropanol. After this, it was washed through cycles of dissolution in toluene, precipitation in isopropanol and centrifugation (20 minutes; 6000 rpm (4427 g)) until the supernatant was clear and transparent. Finally, it was dissolved in toluene and centrifuged at 6000 rpm (4427 g) until precipitation.

#### Quaternization reaction of the CF-PDMAEA (CF-PDMAEA-Q)

First, 0.1 g of CF-PDMAEA was dispersed in 15 mL of DMSO and then heated at 40 °C. Following this, 5 mL (20 mmol) of 1-bromododecane was added and then left to react overnight. Once the reaction was complete, the solid product was recovered by centrifugation (6000 rpm (4427 g), 20 min) and then purified by two cycles of dispersion in DMSO followed by centrifugation (6000 rpm (4427 g), 20 min).

#### Thin-film preparation

Thin films of Cellulose Fiber (CF), CF-PDMAEA, and CF-PDMAEA-Q in PVC were prepared for antibacterial testing as follows:

For PVC film, 2 g of PVC was dissolved by overnight stirring in 30 mL of DMF. The obtained solution was then evaporated under vacuum drying at 50 °C for 3 h.

For films containing fillers such as modified or unmodified fibers or flour, 1.8 g of PVC and 0.2 g of filler were dissolved by overnight stirring in 30 mL of DMF. The obtained solution was then evaporated under vacuum drying at 50 °C for 3 h.

#### Production of the 3D printing filament through the twin-screw extrusion process

Attempts to extrude pure PVC were unsuccessful because of its degradation. In order to process it, the following additives were added:

**Table:** 

PVC	100 g
Mark CZ2000	2.5 g
Paraloid K125 ER	1 g
Loxiol G60	1.2 g
Loxiol G21	0.2 g
Ligalub GT	1.2 g
Licocene PE4201	0.15 g

The mixture was then dried under vacuum overnight and extruded at 145 °C and 35 rpm using counter-rotating screws with a Haake Minilab 3 extruder. In case of clogging of the machine, the screw rotation speed was decreased to 10 rpm. The obtained PVC filaments were white and turned slightly yellow during cooling. Without prior drying, the filaments exhibited much more severe yellowing, reaching a dark orange color, which is attributed to the formation of HCl in the filament as it reacts with moisture, and the subsequent degradation of the polymer. Composite filaments were instead medium brown, with no significant color change observed upon cooling.

#### 3D printing of the produced filament using an FDM printer

The filaments obtained were printed using an Original PRUSA i3 MK3 FDM printer, with the following parameters: nozzle temperature: 210 °C; bed temperature: 90 °C (100 °C for the first layers); printing speed: 5 mm s^−1^; fan speed: off; first layer width: 200%; infill 15%, with gyroid pattern. Printing temperature was increased by 5 °C, and the extrusion multiplier was set to 1.1 to print the composite. Printing speed above 5 mm s^−1^ or lower printing width of the first layers led to high warping and, in the worst cases, complete lack of adherence to the printing bed.

### 2.3. Characterization

^13^C NMR spectra were acquired on a Bruker 500 Avance III HD spectrometer at Larmor frequencies of 125 MHz and 500 MHz for ^13^C and ^1^H, respectively. The samples were put into 4 mm zirconia rotors for magic angle spinning (MAS) at a rotation speed of 8 kHz. Creeping ^13^C MAS NMR spectra were recorded with a ^13^C mutation frequency of 50 kHz and a contact time of 1.5 ms. These spectra were acquired by Fourier transform of the FIDs, and chemical shifts were referenced to pure tetramethylsilane (TMS).

ATR-FTIR (attenuated total reflection Fourier transform infrared spectroscopy) was performed using a Bruker Vector 33 FTIR Fourier-transform infrared spectrometer I18500 PS15. Spectra were recorded with 64 scans in the spectral region of 3800–450 cm^−1^. The spectral resolution is 4 cm^−1^.

For antibacterial testing of thin films, the modified cellulose films were sterilized in 70 vol% ethanol and dried at room temperature before testing their antibacterial activity. Thin films with dimensions of 1 × 1 cm were treated with 2.50 mL diluted bacteria suspension in 15 mL Falcon tubes for 1 h under gentle shaking (60 rpm) at 28 °C for *Pseudomonas aeruginosa* and *Staphylococcus aureus*, and 37 °C for *Escherichia coli*. These films must be completely covered with the bacterial suspension during incubation. The final inoculum size was varied from 10^5^ to 10^8^ CFU mL^−1^ of Gram-negative (*E. coli* K-12 substr. MG1655, *P. aeruginosa* PAO1) and Gram-positive (*S. aureus* ATCC 12600) bacteria. After incubation, an aliquot of 50 µL was plated on Luria–Bertani (LB) agar (Table 1) and incubated for 24 h to 48 h at 28 °C or 37 °C, depending on the bacterial strain. Determination of antibacterial activity of modified cellulose films was performed in triplicate with separately grown cultures.

**Table 1:** Luria-Bertani (LB) composition

NaCl	10 g
Trypregardingn	10 g
Hefeextrakt	5 g
H_2_O_bidest_	Ad 1000 mL

SAXS measurements were performed at the beamline P03 of PETRA III at the DESY synchrotron in Hamburg. Samples were 10 wt% films in PVC, prepared by film casting. The beam wavelength was *λ* = 1023 Å, and the sample-to-detector distance (SDD) was 5441.1 mm. 2D patterns were recorded with a PILATUS 2 M detector (Dectris, Switzerland) with a pixel size of 172 µm. Each measurement was performed by scanning the sample following a 3 × 3 grid with a distance of 1 mm between each point and an acquisition time of 1 s to avoid beam damage. Images were then summed and radially integrated to obtain an *I*(*q*) curve. These curves were fitted using the lmfit Python library and the extended Guinier–Porod model with additional terms for fiber ordering (Lorentzian function) and crystallinity (Gaussian function) (see SI).

Contact angle measurements were performed using a DataPhysics OCA 20 on film-casted samples. No polymer matrix was used.

## 3. Results and discussion

**Scheme 1:**
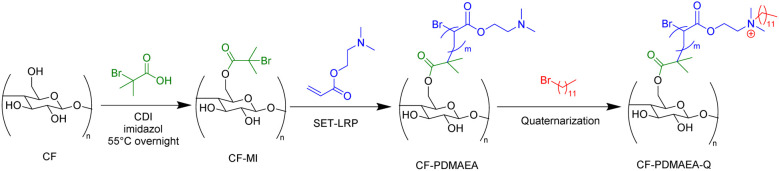
Synthesis scheme for CF-PDMAEAQ.

The first step was to produce cellulose fibers (CF_fiber_) and flour (CF_flour_), transform them into a macroinitiator-based structure (CF-MI), and then initiate the polymerization and graft them with poly(dimethyl dodecyl amino ethyl acrylate) (CF-PDMAEAQ). First, 2-bromoisobutyric acid was grafted on the surface of both cellulose structures (flour and fiber), using CDI and imidazole to promote the reaction (Scheme 1). This surface modification allowed the use of the modified cellulose as a macroinitiator (CF-MI) and initiated the SET-LRP using 2-dimethylaminoethyl acrylate (DMAEA) as a monomer. The product of this reaction (CF-PDMAEA) was then quaternized using 1-iodo-dodecane in order to obtain a combination of cationicity on the nitrogen and hydrophobicity (long alkyl chain), as it is shown to have strong antibacterial properties^49^ (CF-PDMAEAQ).

**Fig. 1:**
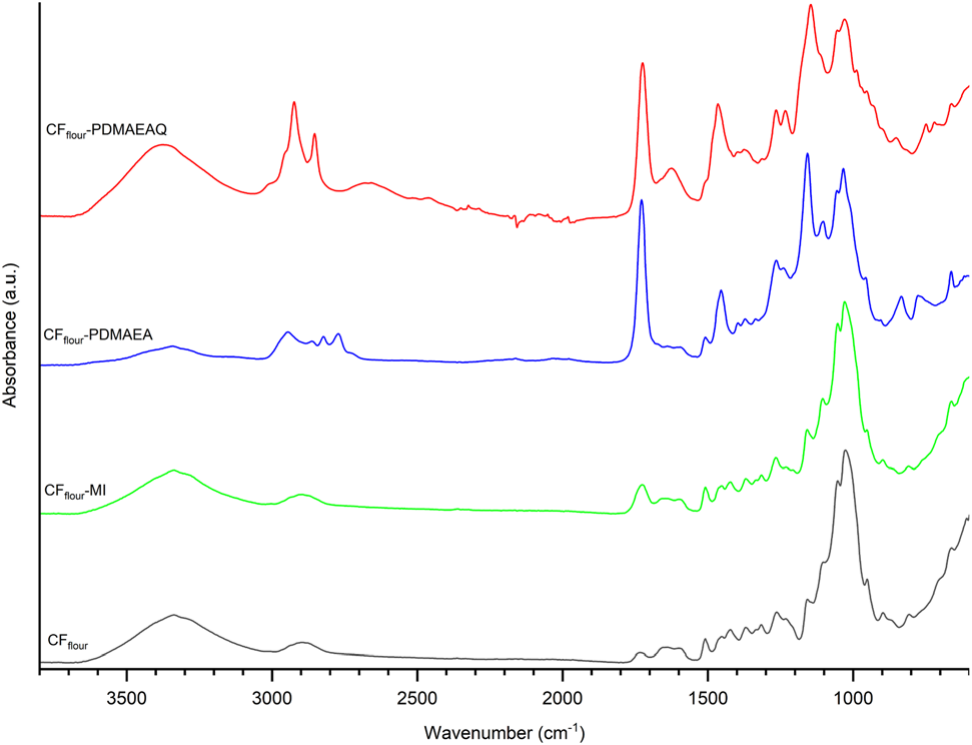
FTIR spectra of cellulose flour (CF_flour_), macro-initiator (CF_flour_-MI), poly-DMAEA-grafted cellulose flour (CF_flour_-PDMAEA), and poly-(quaternized)DMAEA-grafted cellulose flour (CF_flour_-PDMAEAQ).

**Fig. 2:**
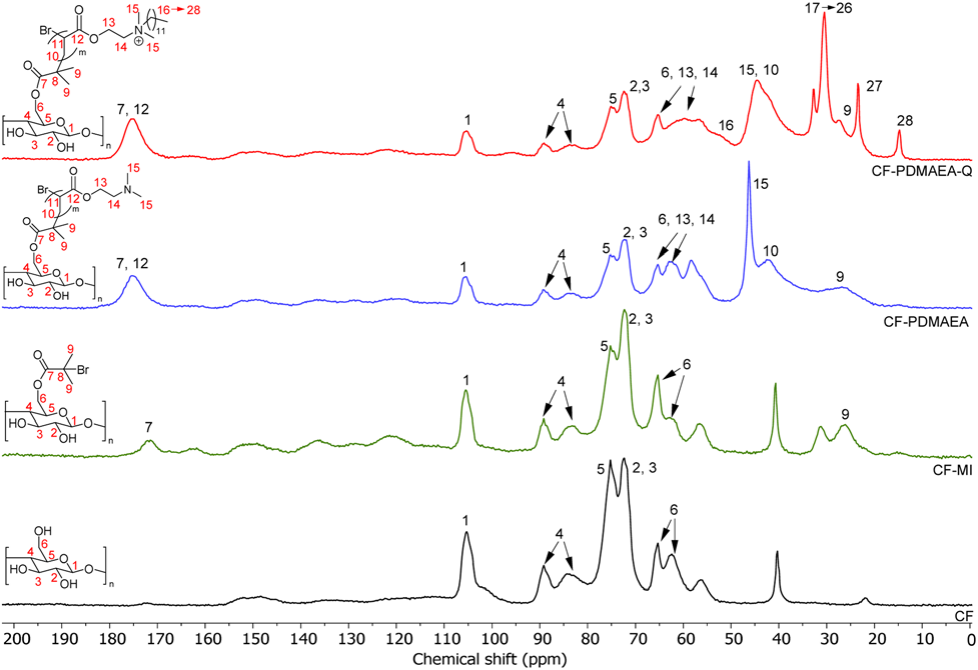
Solid-state ^13^C NMR spectra of (from bottom to top): cellulose flour, macroinitiator, PDMAEA-grafted cellulose fibers, and PDMAEAQ-grafted cellulose flour.

The progress of each step was monitored by FTIR and ^13^C NMR spectroscopy, and the results are presented in Fig. 1 and 2.

The first FTIR spectrum (CF_flour_, in black) in Fig. 1 shows the typical signal for cellulose fibers, such as the O–H stretching band at 3340 cm^−1^ and the C–O stretching band at 1030 cm^−1^. Above it, the spectrum of CF_flour_-MI (in green) shows a similar signal with a higher intensity for the vibration band at 1722 cm^−1^ (carbonyl groups), showing the presence of the ester junction between the cellulose and the initiator. This change in the C=O band shows the success of the first synthesis step.

Regarding the spectra of CF_flour_-PDMAEA (in blue), the absence of the C–H band above 3000 cm^−1^ aside from the –OH band shows that the acrylate's C=C double bond from the monomer is absent from the CF_flour_-PDMAEA product, indicating the absence of the monomer. This confirms that the signals observed come from polymerization and not merely from a physical adsorption of the monomer on the surface of cellulose. In addition to this, the presence of a much stronger C=O band, as well as the appearance of new bands around 1200 and 1450 cm^−1^, are signs that the PDMAEA polymer is indeed present in the product. This confirms that the SET-LPR was successfully performed. Finally, the strong changes in the C–H region around 2900 cm^−1^ in the CF_flour_-PDMAEAQ spectra show the successful addition of the C_12_ carbon chain added for the quaternization. Peak values are given in the SI.

The bottom spectrum in Fig. 2 is the ^13^C NMR spectrum of CF_flour_ (shown in black). One can see the characteristic peaks of cellulose; note that peaks 4 (84 and 89 ppm) and 6 (56 and 62 ppm) are split in two due to the difference in chemical shift between crystalline and amorphous cellulose.^50^ The presence of these peaks in all subsequent spectra allows us to confirm that the original CF_flour_ is present in all other samples.

In the second spectrum (from CF_flour_-MI, in green), one can see the success of the reaction by the appearance of the ester peak at 175 ppm and the CH_3_ peak at 26 ppm. In addition to this, a noticeable change in the shape of the peaks from carbons 6 and 5 demonstrates that the reaction occurred at the C6 hydroxide function. Further changes can be seen on the next spectrum (in blue): the strengthening of the ester peak at 175 ppm and the appearance of the N–CH_3_ peak from carbon 15 at 46 ppm allow us to conclude the presence of reacted monomer in the product. Another important feature of this spectrum is the absence of any peak in the 110–150 ppm region that corresponds to sp^2^ carbons found only in the monomer. This demonstrates the polymerization of DMAEA and the successful removal of unreacted monomer. Together, this information shows the success of the SET-LRP. The peak values are detailed in the SI.

Finally, two key changes can be identified in the last spectrum (green): the first one is the appearance of strong and sharp peaks at low chemical shift (below 40 ppm). These peaks are typical of a long carbon chain, such as the one added by the quaternization of nitrogen by 1-iodododecane. The second major change is the broadening of the peaks corresponding to the carbons 13, 14, and 15. This broadening, together with the conservation of the corresponding area, is attributed to changes in the chemical environment of these carbons caused by the quaternization, as they are the carbons closest to the quaternized nitrogen. This change is seen as a broadening and not a shift because the reaction does not have a 100% yield.

Both FTIR and ^13^C analyses showed the success of all synthesis steps on cellulose flour. The reaction process for cellulose fibers was performed the same way, and no significant differences were found in the spectra. It was therefore concluded that a similar process occurred.

**Fig. 3:**
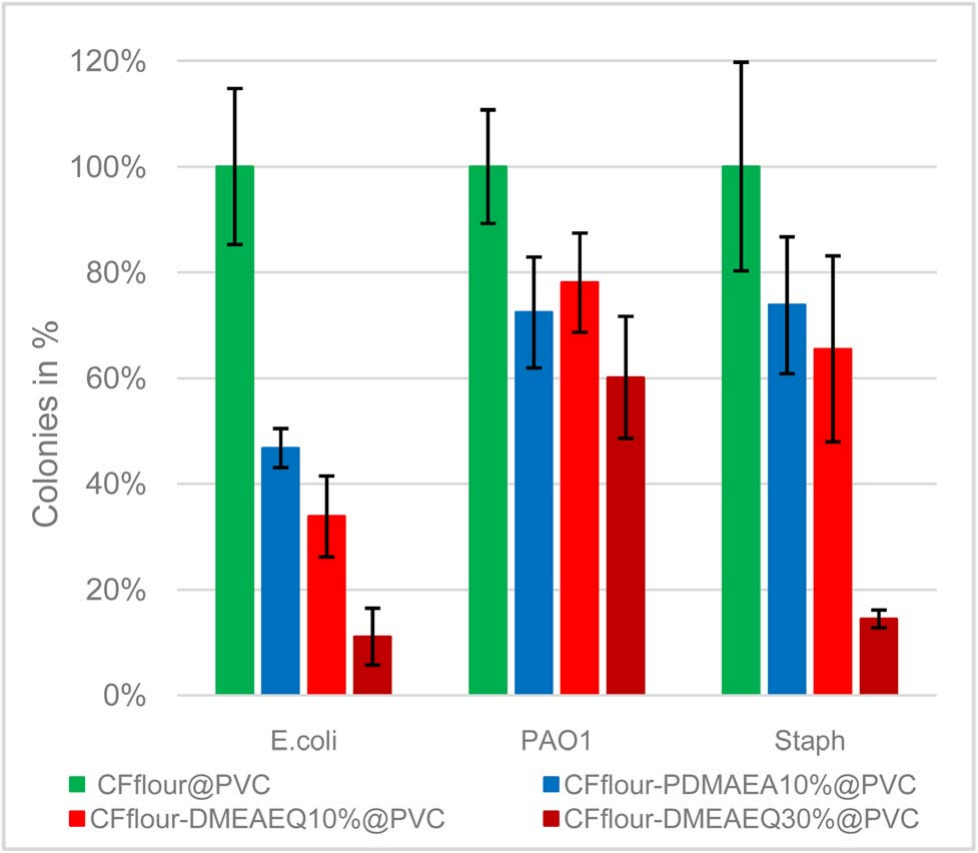
Results from antibacterial testing on various fiber-loaded PVC films on *Escherichia coli* (*E. coli*), *Pseudomonas aeruginosa* (PA01), and *Staphylococcus aureus* (Staph). Films were incubated with 10^3^ cells for 1 h. After treatment time, an aliquot of 50 µL was plated on Luria–Bertani (LB) agar and incubated for 24 h at 28 °C or 37 °C, depending on the bacterial strain.

CF_flour_, CF_flour_-PDMAEA, and CF_flour_-PDMAEAQ were mixed with PVC and made into films by film casting. All films contained 10 wt% of the modified cellulose. An additional sample was made using 30 wt% of CF_flour_-DMAEAQ. The films were then tested using the ASTM E2149-01 standard for antibacterial testing. The results are presented in Fig. 3.

The first thing to be seen is that each modification step increases antibacterial properties. The final product, CF_flour_-PDMAEAQ in PVC, shows some decent antibacterial properties. With 10% loading, it has nearly a 70% reduction in bacterial colonies against *E. coli* (Gram negative) and 35% against *Staphylococcus aureus* (Gram positive). This increases with increased loading, to a 90% reduction against *E. coli* and an 85% reduction against *Staphylococcus aureus*. Results against *Pseudomonas aeruginosa* PAO1 were much lower (only 20% at normal loading, and 40% with increased loading).

These results are consistent with literature reports for similar molecules, in which DMAEA-based polymers exhibited antibacterial activity against both Gram-positive (*S. aureus*) and Gram-negative (*E. coli*) bacteria.^49,51,52^ They also showed higher efficacy against *E. coli* than against *S. aureus*,^51,52^ and substantially lower activity against *P. aeruginosa*.^51^ This shows a good transmission of the antibacterial property from the monomer to the composite.

Regarding the mechanism of action, several mechanisms were advanced to explain the antibacterial properties, one being the fixation of the polymer on the bacteria's membrane through electrostatic interaction followed by the insertion of the long hydrophobic chain through the membrane, causing leakage of the inside constituent of the bacteria and its subsequent death,^49^ while the other mechanism proposed is an inversion of the bacterial membrane's zeta potential due to the polymer's cations, leading to shrinkage of the bacterial membrane and its death.^52^ As the goal of this work was merely to show the successful transmission of the antibacterial properties to the composite, no specific analysis was performed to ascertain the exact action mechanism of this system.

It is to be noted that, while these values are not especially impressive, they come with advantages due to their origins. As the antibacterial properties come from uniformly dispersed, surface-modified cellulose fibers, they are not expected to be vulnerable to surface damage or loss by migration and release of the antibacterial agent, and therefore display damage-resistant and long-lasting antibacterial properties.

**Fig. 4:**
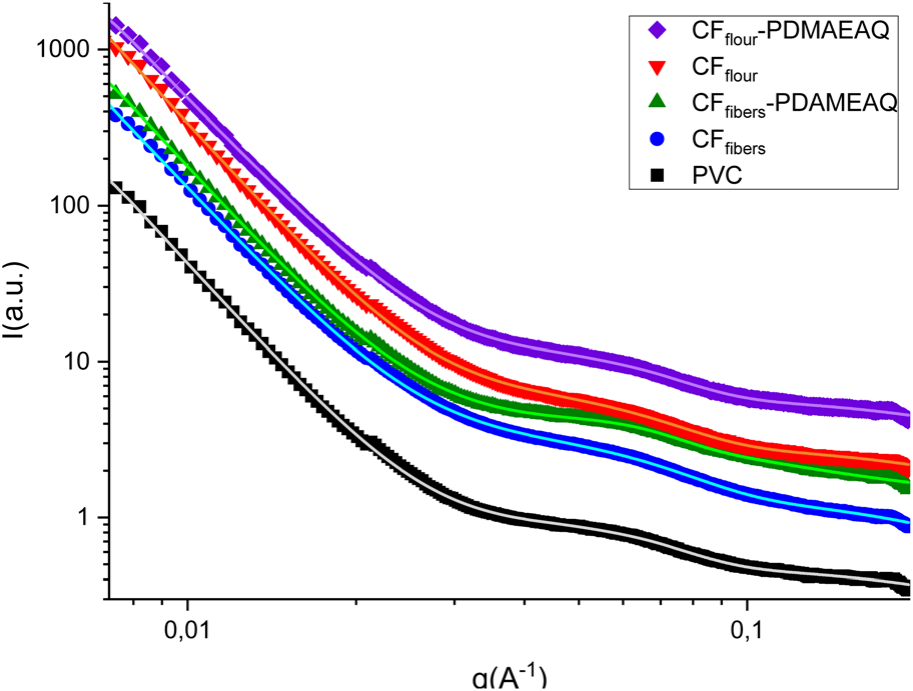
SAXS measurement and their respective fit of (from bottom to top): PVC, CF_fibers_@PVC, CF_fibers_-PDMAEAQ@PVC, CF_flour_@PVC, and CF_flour_-PDMAEAQ@PVC.

After this, samples were measured by SAXS (Fig. 4). The samples were pure PVC; PVC with 10 wt% in cellulose flour (CF_flour_@PVC); PVC with 10 wt% of cellulose flour grafted with DMAEAQ (CF_flour_-PDMAEAQ_10%_@PVC); PVC with 10 wt% in cellulose fiber (CF_fibers_@PVC); and PVC with 10 wt% of cellulose fiber grafted with DMAEAQ (CF_fibers_-PDMAEAQ_10%_@PVC).

Although little difference can be observed in the SAXS spectra of the samples, the fitting values show some key changes: the Porod exponent of the PVC sample has a value of 3.66, while it decreases to 3.51 for CF_flour_@PVC, and to 3.56 for CF_fiber_@PVC. This was attributed to the fact that cellulose microfibrils inside the cellulose fibres are more unfolded than PVC, their contribution lowering the overall Porod exponent of the sample. This also shows that cellulose fibres, due to their smaller size compared to cellulose flour, have a weaker effect on the overall signal.

In addition to this, the Porod exponent further decreases to 3.48 for CF_flour_-PDMAEAQ_10%_@PVC, while it increases to 3.62 for CF_fiber_-PDMAEAQ_10%_@PVC. This shows 2 things: first, the polymers grafted on the surface of cellulose are bundled in sphere-like structures, as opposed to having unfolded chains. This can be seen by the increase in the Porod exponent with the grafting of polymer on the surface of cellulose fibres. With cellulose flour, however, the Porod exponent decreases compared to unmodified flour instead. This was attributed to a better mixing of the cellulose with the matrix, which increases the impact of cellulose on the signal, and to the larger size of cellulose compared to the fibres, which makes the cellulose's effect on the signal more important than that of the grafted polymer. This better mixing was seen as proof of increased fibre–matrix compatibility.

**Fig. 5:**
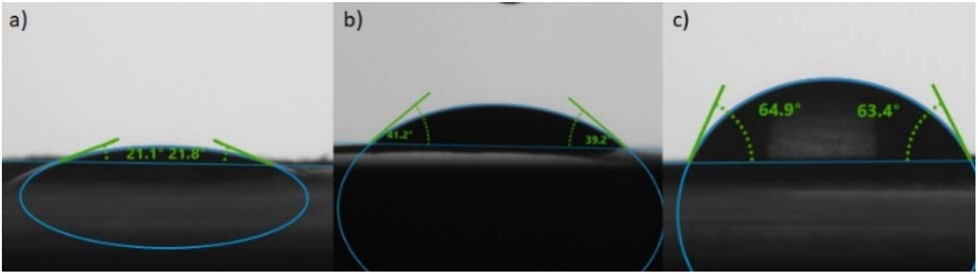
Contact angle measurements of (a) CF_fibers_ film, (b) CF_fibers_-PDMAEAQ film, and (c) PVC film.

Then, the contact angles were measured (Fig. 5). Only the values for fibers and modified fibers were measured, as cellulose flour was not expected to form a film.

Unmodified cellulose fibers' contact angle was hard to measure, as the water drop was absorbed by the film most of the time, finally yielding a value of 21°. CF_fibers_-PDMAEAQ had a contact angle value of 40°, and PVC had a contact angle value of 60°.

While the contact angle of PVC remained higher than that of modified cellulose, the contact angle difference was reduced by half compared to CF_fibers_. CF_fibers_-PDMAEAQ exhibited sufficiently low porosity and hydrophilicity to allow reliable contact angle measurements. This reduced difference in hydrophobicity between PVC and the modified fibers explains the increased compatibility observed by SAXS, and highlights another advantage of this treatment, in addition to its antibacterial properties.

**Fig. 6:**
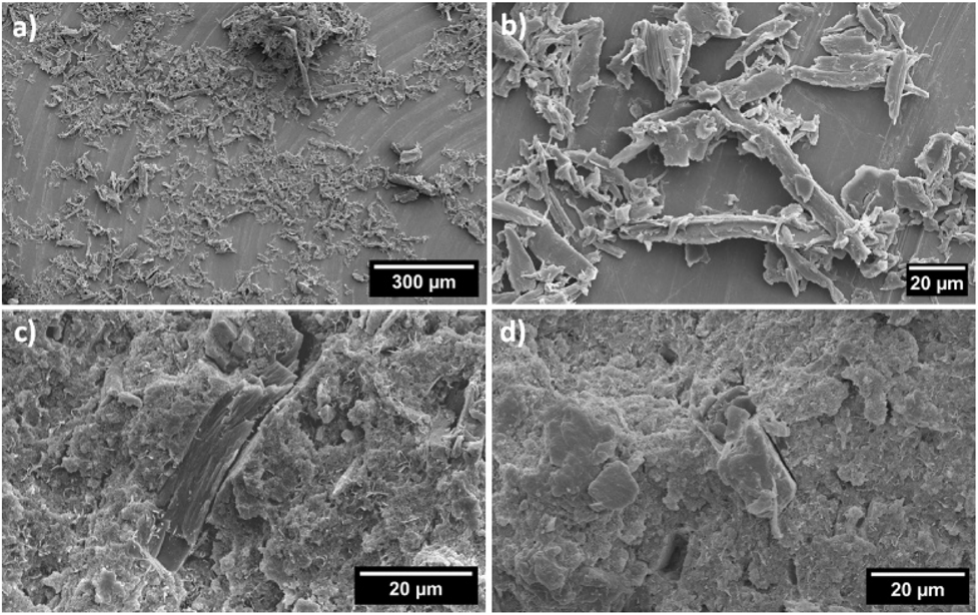
FESEM pictures of CF_flour_ (pictures (a) and (b)) and broken CF_flour_-PDMAEAQ_10%_@PVC composite (pictures (c) and (d)).

Finally, filaments of PVC and CF_flour_-PDMAEAQ_10%_@PVC were made by extrusion for 3D printing. This time, additives were added to PVC to make it processable by extrusion. The compounding temperature was fixed at 145 °C, in order to avoid filament degradation. For the same reason, both the extruder and the PVC were dried before compounding. Before printing, the tip of the filament was broken, and the broken section was observed by FESEM (Fig. 6).

The FESEM pictures show a mixed result in the matter of cellulose-matrix compatibility: while Fig. 6d shows a pull-out and both Fig. 6c and d show a clear demarcation between the cellulose flour and the PVC matrix, the fact that most of the cellulose flour remained in the matrix and that no cellulose flour clusters were observed indicated a better compatibility than unmodified cellulose. These observations are in accordance with the increased proximity between the contact angle values of PVC and CF_fibers_-PDMAEAQ compared to CF_fibers_.

**Fig. 7:**
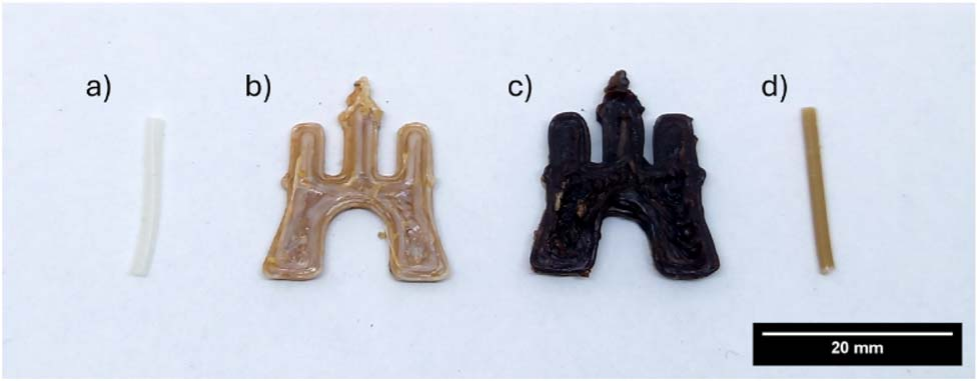
Picture of (from right to left): (a) PVC filament, (b) PVC print, (c) CF_flour_-DAMAEAQ_10%_@PVC print, and (d) CF_flour_-PDAMAEAQ_10%_@PVC filament.

Once the filaments were made, both PVC and CF_flour_-PDMAEAQ_10%_@PVC could be printed (Fig. 7). While CF_flour_-PDAMAEAQ filaments needed a higher nozzle temperature and extrusion multiplier (the change was similar to the change made between commercial PLA filament and commercial woodfill PLA filament). The printing of CF_flour_-PDMAEAQ_10%_@PVC led to a lower quality of print than PVC, because, partly due to over-extrusion and extensive degradation of the modified cellulose within the filament during printing, as evidenced by the color change of the composite and smoke evolution during printing.

Seeing the high degradation of the CF_flour_-PDMAEAQ_10%_@PVC filament, it was deemed unnecessary to print the CF_fiber_-PDMAEAQ_10%_@PVC filament, as it would undergo the same degradation for the same reason.

This degradation problem was hard to correct with PVC as the matrix because PVC needs to be printed at temperatures that also lead to CF-PDMAEAQ degradation, while also being very sensitive to the high temperature needed to print it. In addition to that, the requirement of a very low printing speed for PVC (5 mm s^−1^ instead of 30 mm s^−1^ for other polymers) led to increased degradation of the CF_flour_-PDAMEAQ fibers and of PVC itself. This restriction is especially significant with CF-PDAMAEAQ, as, while a short time at this temperature would minimize degradation, slow print speed ensures a long heating time and maximizes the degradation of the composite. While it remains printable, the use of PVC as a matrix results in much lower printability. On the other hand, other matrices are expected to provide much better print quality, as they would not undergo degradation during the printing process and could enable higher printing speeds, thereby improving the preservation of the cellulose fibers.

As the degradation of the grafted cellulose in PVC indicated that this cellulose modification/polymer matrix pair was not optimal, and because the extent of cellulose degradation indicated that no improvement in composite's mechanical properties could be expected, mechanical testing was not performed.

## 4. Conclusions

In this study, we modified cellulose flour and cellulose fiber by using SET-LRP to graft quaternized 2-dimethylammonium ethyl acrylate. The operation was successful, and the CF-PDMAEAQ@PVC composite showed antibacterial activity. Better compatibility between the matrix and CF-PDMAEAQ was observed when compared to unmodified cellulose fibers, as evidenced by the contact angle values of modified *versus* unmodified fiber and PVC. Finally, the printability of the composite was tested, showing that, while the addition of CF-PDMAEAQ to the matrix posed some challenges, the resulting composite remained printable. While having good compatibility with CF-PDAMAEAQ, PVC was shown to be bad for printing, exacerbating the challenges associated with the CF-PDMAEAQ-loaded composite. A promising continuation of this study would be to change the matrix to a more easily printed one, such as PLA.

## Author contributions

The manuscript was written through the contributions of all authors. All authors have approved the final version of the manuscript.

## Conflicts of interest

The authors declare that they have no conflict of interest regarding the content of this article.

## Supplementary Material

RA-OLF-D6RA03699F-s001

## Data Availability

The data supporting this article have been included as part of the supplementary information (SI). Supplementary information: equation and fitting values for the SAXS fitting, FTIR and NMR main peaks and interpretations. See DOI: https://doi.org/10.1039/d6ra03699f.
