# Supplementary material for: Antibacterial cellulose composite: using SET-LRP for cellulose surface modification with quaternized poly-dimethylammoniumethylacrylate
Source: RSC Adv. 2026 Jul 8;16(36):38579–87. doi: 10.1039/d6ra03699f (PMC13343542; doi:10.1039/d6ra03699f)
Supplement: Supplementary file 1 [file RA-016-D6RA03699F-s001.pdf]

Supplementary Information (SI) for RSC Advances.
This journal is © The Royal Society of Chemistry 2026

Production of an Antibacterial FDM 3D-printing filament made through the compounding of polymer grafted cellulose fibers and PVC

Enguerrand Barba[a], J. Benedikt Mietner[a], Dhanya Raveendran[a], Benedikt Sochor[b,c], Sarathlal Koyiloth Vayalil[b,d], Calvin Tu[e], Christel Vollstedt[e], Wolfgang Streit[e], Stephan V. Roth[b,f], Julien R.G. Navarro[*,a]

Supplementary information:

1. SAXS Fitting: 2
2. SAXS Fitting values 3
PVC: 3
CF_{flour}@PVC 4
CF_{flour}-PDMAEA$Q_{10\%}$@PVC 4
CF_{fibre}-PDMAEA$Q_{10\%}$@PVC 6
3. IR peaks: 6
Cellulose[3–5]: 6
Modified Cellulose: 7
4. NMR peaks: 7
Cellulose: 7
Macro-Initiator: 7
CF-PDMAEA: 8
CF-DPMAEAQ: 8
5. References: 8

1. SAXS Fitting:

Fit was done using the extended Guinier-Porod model[1] with contributions for a gel[2]:

$$I(q) = F(q) + I_g e^{-\frac{(q-q_0)^2\theta^2}{2}} + \frac{I_l}{1+q^2\xi^2} + B$$

With F(q) being the extended Guinier-Porod model:

$$F(q) = \frac{I_0}{q^s} e^{-\frac{x^2 R_g^2}{3-s}}$$

for q < q_1, otherwise:

$$F(q) = Dq^{-n}$$

With:

$$q_1 = \frac{1}{R_g}\left(\frac{(3-s)(n-s)}{2}\right)^{1/2}$$

$$D = I_0 e^{-\frac{q_1^2 R_g^2}{3-s}} q_0^{(n-s)}$$

for continuity.

n is the Porod exponent, indicative of the particles' shapes

$\theta = \frac{1}{\sigma}$ with σ the variance of a Gaussian function, indicative of the size of aggregates

$\xi = \frac{1}{\gamma}$ with γ the scale factor of the Lorentz distribution, indicative of the correlation distance between adjacent fibers.

B is a flat background

Additional information on the crystallite size were calculated using the Debye-Scherrer equation: $D = \frac{K\lambda}{\beta \cos(B)}$

With D: crystallite size

K: Scherrer constant (0.98)

λ: wavelength of beam (=1.048 A)

β: FWHM (radians) : $FWMH = \frac{2\sqrt{2\ln(2)}}{\theta} = \left(x_0 + \frac{\sqrt{2\ln(2)}}{\theta}\right) - \left(x_0 - \frac{\sqrt{2\ln(2)}}{\theta}\right)\ (in\ q)$

$$FWHM = \sin^{-1}\left(\frac{\left(x_0 + \frac{\sqrt{2\ln(2)}}{\theta}\right)\lambda}{4\pi}\right) - \sin^{-1}\left(\frac{\left(x_0 - \frac{\sqrt{2\ln(2)}}{\theta}\right)\lambda}{4\pi}\right)$$ (in radian)

B: Bragg angle

And converting q in radian:

$$B = \sin^{-1}\left(\frac{q\lambda}{4\pi}\right)$$

Therefore:

$$D = \frac{K\lambda}{\left(\sin^{-1}\left(\frac{\left(x_0 + \frac{\sqrt{2\ln(2)}}{\theta}\right)\lambda}{4\pi}\right) - \sin^{-1}\left(\frac{\left(x_0 - \frac{\sqrt{2\ln(2)}}{\theta}\right)\lambda}{4\pi}\right)\right)\cos\left(\sin^{-1}\left(\frac{x_0\lambda}{4\pi}\right)\right)}$$

The peak sizes were corrected using the calibration measurements from the SAXS line:
$\beta_{corrected} = \sqrt{\beta_{fit}^{2} - \beta_{calibration}^{2}}$

The following values were obtained from fitting:

	PVC	CFs@PVC	CFs-DMAEAQ@PVC		PVC	CFl@PVC	CFl-DMAEAQ@PVC
n	3.66	3.51	3.48		3.66	3.56	3.62
x0	0.048	0.048	0.046		0.048	0.048	0.049
Crystallite size ($\frac{2\pi}{x_0}$)	131	131	137		131	131	127
θ	45	45	43		45	45	47
Intercrystallite distance (Debye-Scherrer)	237	136	223		237	237	246
Ξ	2.8	2.8	2.8		2.8	2.9	2.9

2. SAXS Fitting values

After an initial estimation of the fitting values with a python program using the lmfit library, SASView was used for more accurate fitting, and to obtain the uncertainty on the fitted values. The background value of the composite sample was fixed to the value obtained for PVC alone. Finally, the individual contribution of the Guinier -Porod model, the Lorentzian and the Gaussian were plotted to estimate whether or not each part of the model had enough impact on the final curve to be considered relevant.
The fitted results obtained were the following:

PVC:

**Variable**	**Value**	**Error**
$\mathbf{I_0}$	34	81
$\mathbf{R_g}$	318	49
**s**	0.4	0.42
**n**	3.664	0.001
$\mathbf{I_g}$	0.1040	0.0009
$\mathbf{x_0}$	0.0479	0.0001
**σ**	0.0221	0.0001
$\mathbf{I_l}$	0.16	0.03
ξ	2.8	0.4
**B**	0	0.034

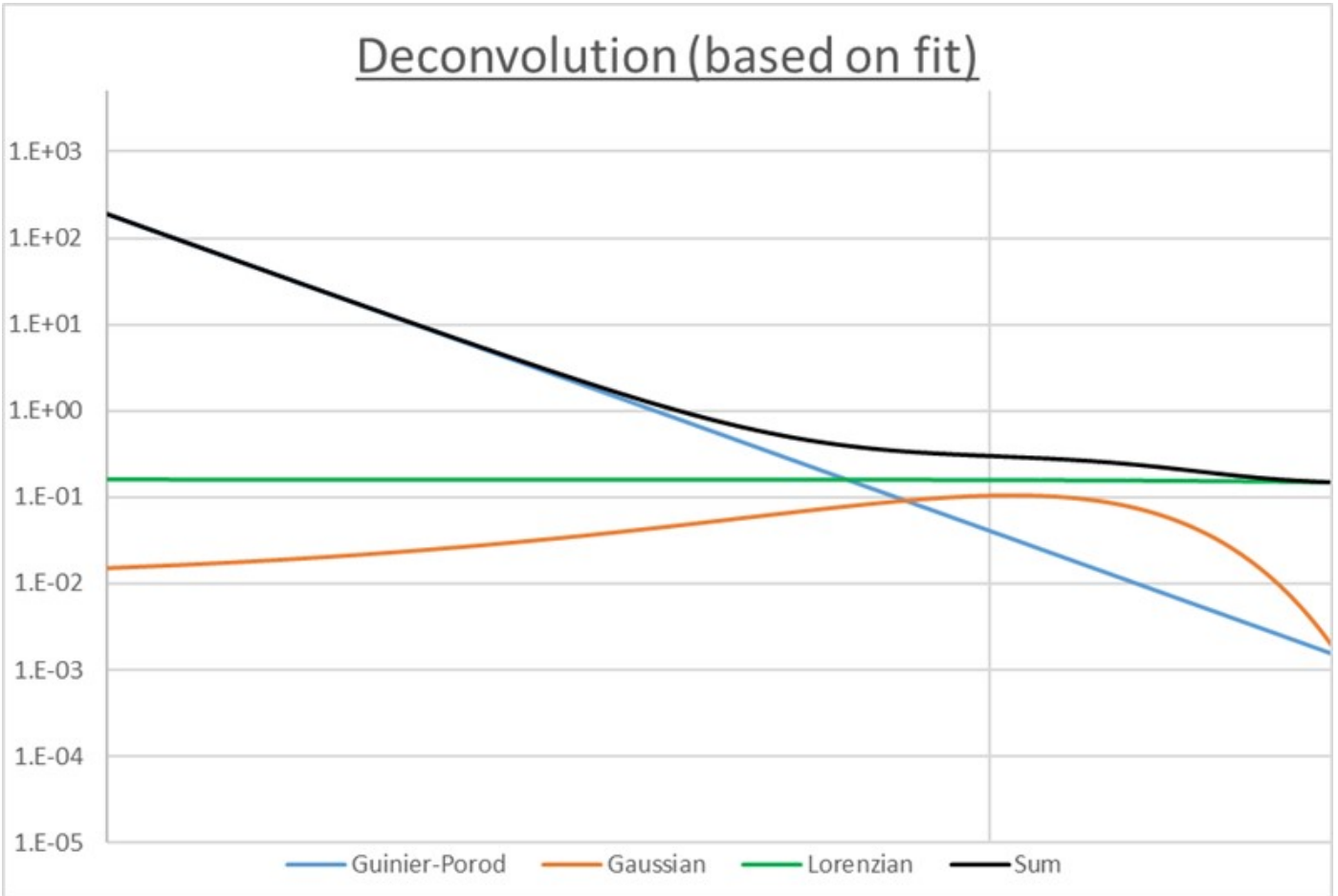

CF$_{flour}$@PVC

Variable	Value	Error
I_0	26	31
R_g	301	25
s	0.42	0.22
n	3.51	0.001
I_g	0.119	0.0007
x_0	0.0475	7.94 10^{-5}
σ	0.0222	0.0001
I_l	0.19	0.03
ξ	2.79	0.31
B	0	-

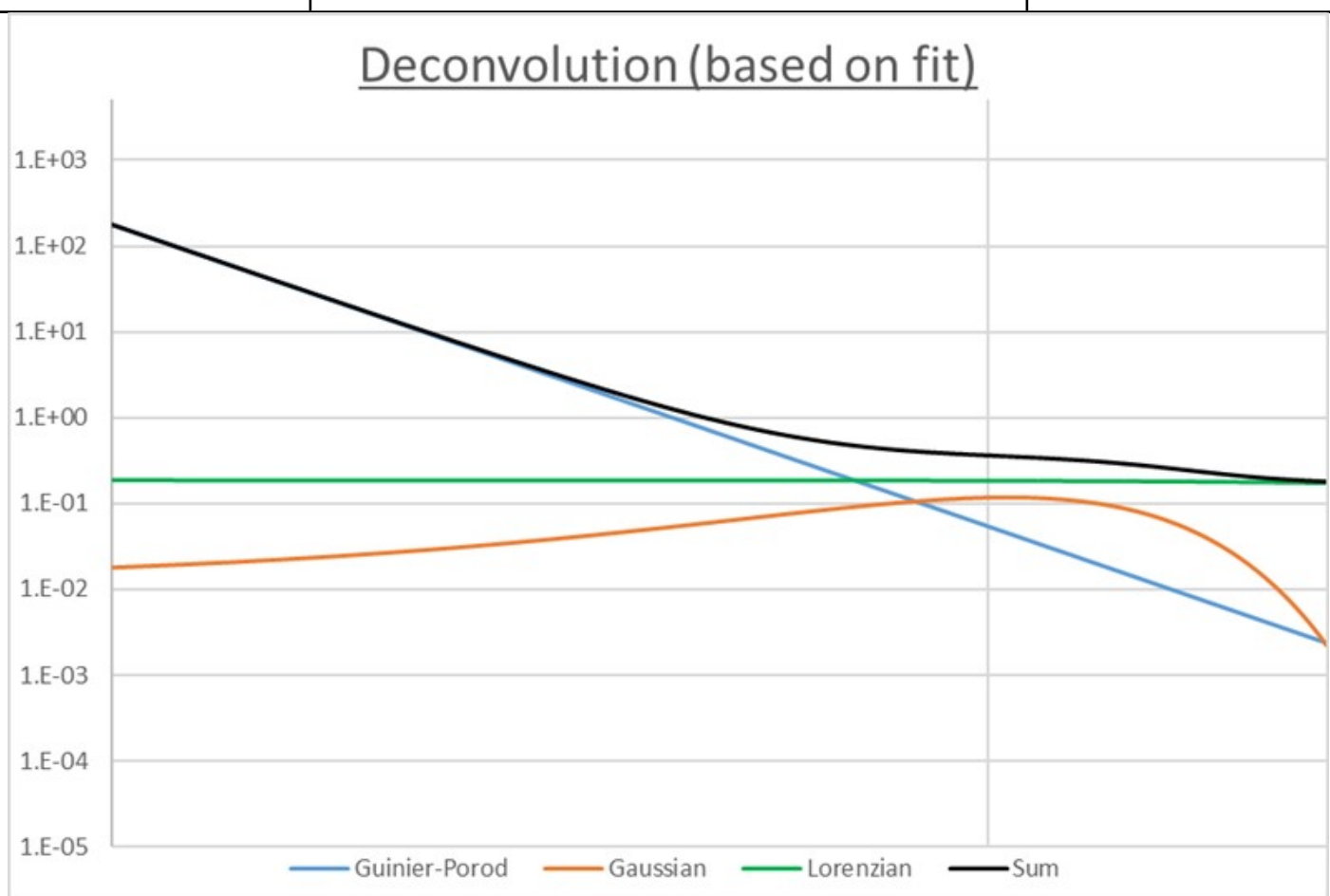

CF$_{flour}$-PDMAEAQ$_{10\%}$@PVC

Variable	Value	Error
I_0	42	136
R_g	322	67
s	0.36	0.57
n	3.48	0.001
I_g	0.1177	0.0003
x_0	0.0459	8.7 10^{-5}

σ	0.0234	8.7 10^{-5}
I_l	0.1803	0.0003
ξ	2.8	0.02
B	0	-

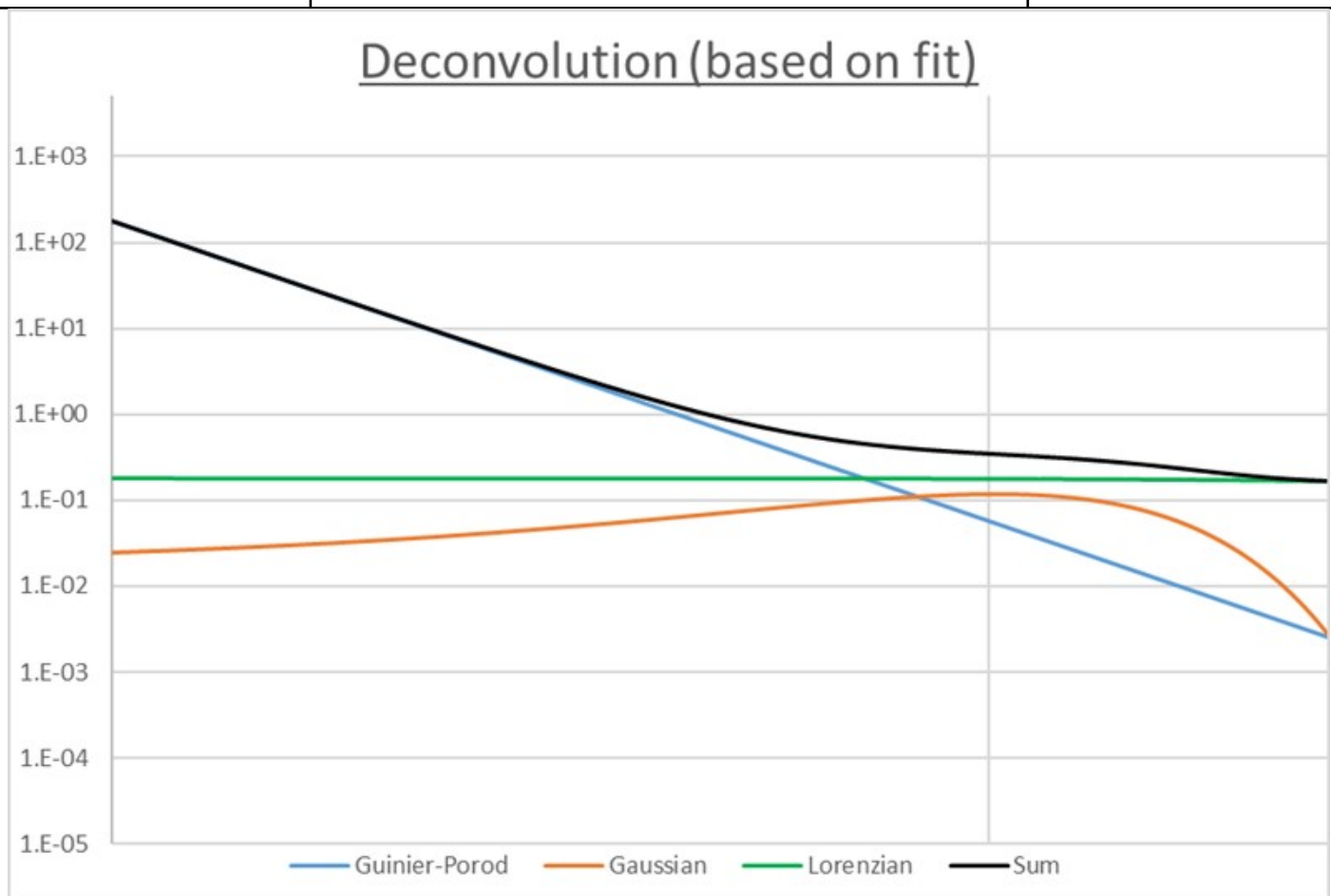

CF_{fibre}@PVC

Variable	Value	Error
I_0	20	24
R_g	300	24
s	0.45	0.21
n	3.56	0.0009
I_g	0.127	0.0002
x_0	0.0482	5.27 10^{-5}
σ	0.0221	5.78 10^{-5}
I_l	0.201	0.0003
ξ	2.88	0.01
B	0	-

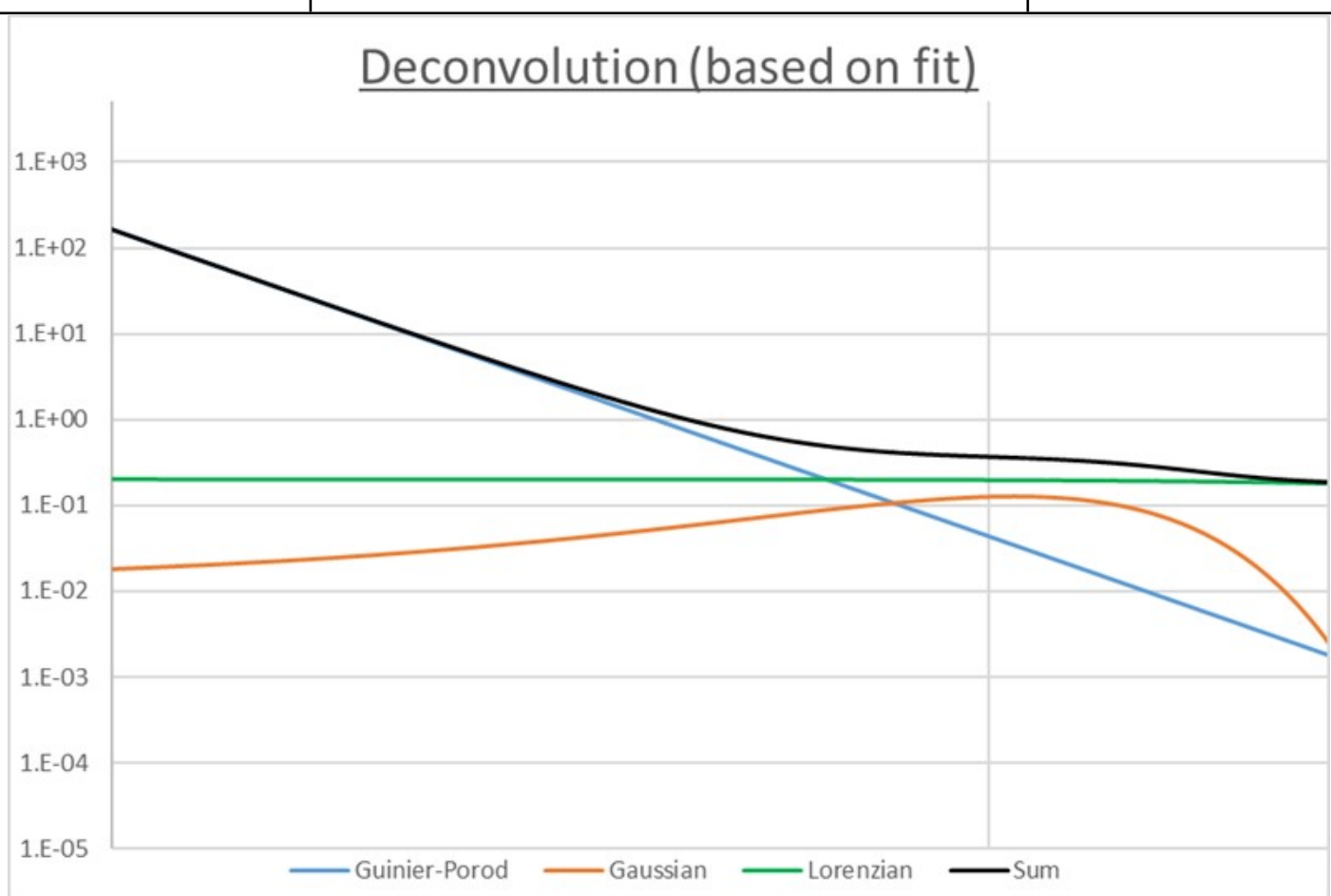

CF_{fibre}-$PDMAEAQ_{10\%}$@PVC

Variable	Value	Error
I_0	12.7	16
R_g	291	26
s	0.55	0.23
n	3.621	0.001
I_g	0.1118	0.0003
x_0	0.0494	$6.3\ 10^{-5}$
σ	0.0213	$7.2\ 10^{-5}$
I_l	0.1794	0.0003
ξ	2.93	0.015
B	0	-

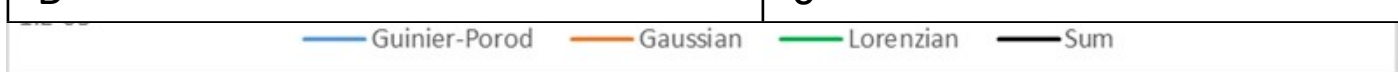

3. IR peaks:

The following values are the peak values obtained from the IR analysis, and their respective attributions:

Cellulose[3–5]:

Wave number (cm^{-1})	Attribution
3340	O-H stretching
2870 - 2930	C-H stretching
1590 - 1650	Absorbed H_2O
1160	In-ring C-O-C stretching
1030	C-O stretching

Modified Cellulose:

Wave number (cm^{-1})	Attribution
2950	C-H stretching
2930	
2850	
2830	
1770	
1722	C=O stretching
1460	
1160	C-N stretching

4. NMR peaks:

The following values are the peak values obtained from the MNR analysis, and their respective attributions:

Cellulose:

Chemical displacement (ppm)	Attribution
40	impurities
56	impurities
62	6
65	6
72	2,3
75	5
84	4
89	4
101	impurities
105	1

Macro-Initiator:

Chemical displacement (ppm)	Attribution
26	9
31	impurities
41	impurities
57	impurities
62	6
65	6
72	2,3
75	5
84	4
89	4
105	1
172	7

CF-PDMAEA:

Chemical displacement (ppm)	Attribution
27	9
42	10
46	15
58	impurities
62	6,13,14
65	6,13,14
72	2,3
75	5
84	4
89	4
105	1
174	1,12

CF-DPMAEAQ:

Chemical displacement (ppm)	Attribution
14	28
23	27
27	8
31	17→26
33	impurities
44	15,10
56	impurities
60	6,13,14
65	6,13,14
72	2,3
75	5
84	4
89	4
105	1
175	7,12

Since the cellulose flour used contained traces of lignin, impurities were responsible for some peaks seen in NMR.

5. References:

1 B. Hammouda, A new Guinier–Porod model, *J Appl Crystallogr*, 2010, **43**, 716–719.

2 V. Guccini, S. Yu, M. Agthe, K. Gordeyeva, Y. Trushkina, A. Fall, C. Schütz and G. Salazar-Alvarez, Inducing nematic ordering of cellulose nanofibers using osmotic dehydration, *Nanoscale*, 2018, **10**, 23157–23163.

3 E. Singovszka, Characterization of Cellulosic Fibers by FTIR Spectroscopy for Their Further Implementation to Building Materials, *American Journal of Analytical Chemistry*, DOI:10.4236/AJAC.2018.96023.

4 C. Trilokesh and K. B. Uppuluri, Isolation and characterization of cellulose nanocrystals from jackfruit peel, *Sci Rep*, 2019, **9**, 16709.

5 O. S. Samuel, A. M. Adefusika, O. S. Samuel and A. M. Adefusika, in *Cellulose*, IntechOpen, 2019.
