# Supplementary material for: Antibacterial cellulose composite: using SET-LRP for cellulose surface modification with quaternized poly-dimethylammoniumethylacrylate
Source: RSC Adv. 2026 Jul 8. Online ahead of print. doi: 10.1039/d6ra03699f (PMC13343542; doi:10.1039/d6ra03699f)
Supplement: RA-OLF-D6RA03699F-s001 [file RA-OLF-D6RA03699F-s001.pdf]

Supplementary Information (SI) for RSC Advances.
This journal is © The Royal Society of Chemistry 2026

# Production of an Antibacterial FDM 3D-printing filament made through the compounding of polymer grafted cellulose fibers and PVC

Enguerrand Barba[a], J. Benedikt Mietner[a], Dhanya Raveendran[a], Benedikt Sochor[b,c], Sarathlal Koyiloth Vayalil[b,d], Calvin Tu[e], Christel Vollstedt[e], Wolfgang Streit[e], Stephan V. Roth[b,f], Julien R.G. Navarro[*,a]

## Supplementary information:

1. SAXS Fitting: 2
2. SAXS Fitting values 3
PVC: 3
$CF_{flour}$@PVC 4
$CF_{flour}$-$PDMAEAQ_{10\%}$@PVC 4
$CF_{fibre}$-$PDMAEAQ_{10\%}$@PVC 6
3. IR peaks: 6
Cellulose[3–5]: 6
Modified Cellulose: 7
4. NMR peaks: 7
Cellulose: 7
Macro-Initiator: 7
CF-PDMAEA: 8
CF-DPMAEAQ: 8
5. References: 8

# 1. SAXS Fitting:

Fit was done using the extended Guinier-Porod model[1] with contributions for a gel[2]:

$$I(q) = F(q) + I_g e^{-\frac{(q-q_0)^2\theta^2}{2}} + \frac{I_l}{1+q^2\xi^2} + B$$

With F(q) being the extended Guinier-Porod model:

$$F(q) = \frac{I_0}{q^s} e^{-\frac{x^2R_g^2}{3-s}}$$

for q < $q_1$, otherwise:

$$F(q) = Dq^{-n}$$

With:

$$q_1 = \frac{1}{R_g}\left(\frac{(3-s)(n-s)}{2}\right)^{1/2}$$

$$D = I_0 e^{-\frac{q_1^2R_g^2}{3-s}} q_0^{(n-s)}$$

for continuity.

n is the Porod exponent, indicative of the particles' shapes

$\theta = \frac{1}{\sigma}$ with σ the variance of a Gaussian function, indicative of the size of aggregates

$\xi = \frac{1}{\gamma}$ with $\gamma$ the scale factor of the Lorentz distribution, indicative of the correlation distance between adjacent fibers.

B is a flat background

Additional information on the crystallite size were calculated using the Debye-Scherrer equation: $D = \frac{K\lambda}{\beta\cos(B)}$

With D: crystallite size

K: Scherrer constant (0.98)

λ: wavelength of beam (=1.048 A)

β: FWHM (radians) : $FWMH = \frac{2\sqrt{2\ln(2)}}{\theta} = \left(x_0 + \frac{\sqrt{2\ln(2)}}{\theta}\right) - \left(x_0 - \frac{\sqrt{2\ln(2)}}{\theta}\right)$ $(in\ q)$

$$FWHM = \sin^{-1}\left(\frac{\left(x_0 + \frac{\sqrt{2\ln(2)}}{\theta}\right)\lambda}{4\pi}\right) - \sin^{-1}\left(\frac{\left(x_0 - \frac{\sqrt{2\ln(2)}}{\theta}\right)\lambda}{4\pi}\right)$$ (in radian)

B: Bragg angle

And converting q in radian:

$$B = \sin^{-1}\left(\frac{q\lambda}{4\pi}\right)$$

Therefore:

$$D = \frac{K\lambda}{\left(\sin^{-1}\left(\frac{\left(x_0 + \frac{\sqrt{2\ln(2)}}{\theta}\right)\lambda}{4\pi}\right) - \sin^{-1}\left(\frac{\left(x_0 - \frac{\sqrt{2\ln(2)}}{\theta}\right)\lambda}{4\pi}\right)\right)\cos\left(\sin^{-1}\left(\frac{x_0\lambda}{4\pi}\right)\right)}$$

The peak sizes were corrected using the calibration measurements from the SAXS line:
$\beta_{corrected} = \sqrt{\beta_{fit}^2 - \beta_{calibration}^2}$

The following values were obtained from fitting:

| | PVC | CFs@PVC | CFs-DMAEAQ@PVC | | PVC | CFl@PVC | CFl-DMAEAQ@PVC |
|---|---|---|---|---|---|---|---|
| n | 3.66 | 3.51 | 3.48 | | 3.66 | 3.56 | 3.62 |
| x0 | 0.048 | 0.048 | 0.046 | | 0.048 | 0.048 | 0.049 |
| Crystallite size ($\frac{2\pi}{x_0}$) | 131 | 131 | 137 | | 131 | 131 | 127 |
| $\theta$ | 45 | 45 | 43 | | 45 | 45 | 47 |
| Intercrystallite distance (Debye-Scherrer) | 237 | 136 | 223 | | 237 | 237 | 246 |
| Ξ | 2.8 | 2.8 | 2.8 | | 2.8 | 2.9 | 2.9 |

## 2. SAXS Fitting values

After an initial estimation of the fitting values with a python program using the lmfit library, SASView was used for more accurate fitting, and to obtain the uncertainty on the fitted values. The background value of the composite sample was fixed to the value obtained for PVC alone. Finally, the individual contribution of the Guinier -Porod model, the Lorentzian and the Gaussian were plotted to estimate whether or not each part of the model had enough impact on the final curve to be considered relevant.
The fitted results obtained were the following:

PVC:

| **Variable** | **Value** | **Error** |
|---|---|---|
| $\mathbf{I_0}$ | 34 | 81 |
| $\mathbf{R_g}$ | 318 | 49 |
| **s** | 0.4 | 0.42 |
| **n** | 3.664 | 0.001 |
| $\mathbf{I_g}$ | 0.1040 | 0.0009 |
| $\mathbf{x_0}$ | 0.0479 | 0.0001 |
| **σ** | 0.0221 | 0.0001 |
| $\mathbf{I_l}$ | 0.16 | 0.03 |
| $\xi$ | 2.8 | 0.4 |
| **B** | 0 | 0.034 |

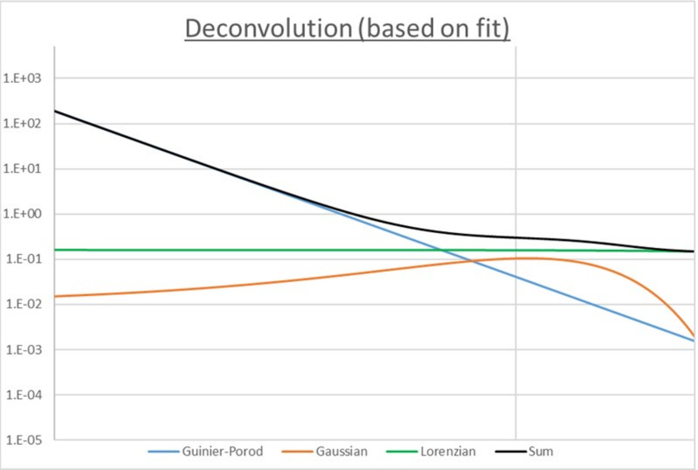


$CF_{flour}$@PVC

| Variable | Value | Error |
|---|---|---|
| $I_0$ | 26 | 31 |
| $R_g$ | 301 | 25 |
| s | 0.42 | 0.22 |
| n | 3.51 | 0.001 |
| $I_g$ | 0.119 | 0.0007 |
| $x_0$ | 0.0475 | $7.94\ 10^{-5}$ |
| σ | 0.0222 | 0.0001 |
| $I_l$ | 0.19 | 0.03 |
| ξ | 2.79 | 0.31 |
| B | 0 | - |

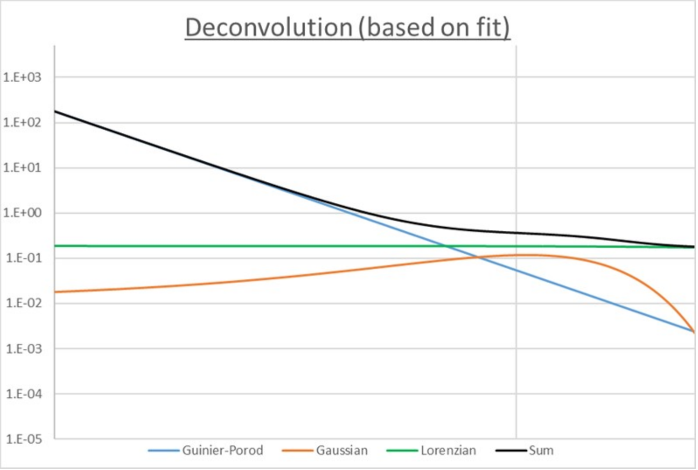


$CF_{flour}$-$PDMAEAQ_{10\%}$@PVC

| Variable | Value | Error |
|---|---|---|
| $I_0$ | 42 | 136 |
| $R_g$ | 322 | 67 |
| s | 0.36 | 0.57 |
| n | 3.48 | 0.001 |
| $I_g$ | 0.1177 | 0.0003 |
| $x_0$ | 0.0459 | $8.7\ 10^{-5}$ |

| | | |
|---|---|---|
| σ | 0.0234 | 8.7 $10^{-5}$ |
| $I_l$ | 0.1803 | 0.0003 |
| ξ | 2.8 | 0.02 |
| B | 0 | - |

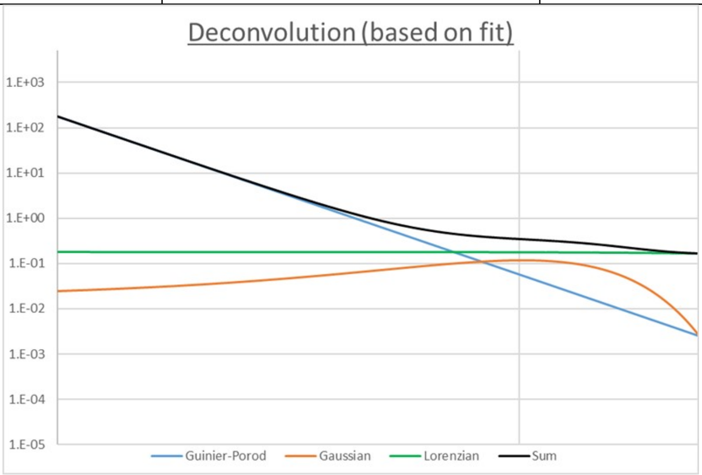


$CF_{fibre}$@PVC

| Variable | Value | Error |
|---|---|---|
| $I_0$ | 20 | 24 |
| $R_g$ | 300 | 24 |
| s | 0.45 | 0.21 |
| n | 3.56 | 0.0009 |
| $I_g$ | 0.127 | 0.0002 |
| $x_0$ | 0.0482 | 5.27 $10^{-5}$ |
| σ | 0.0221 | 5.78 $10^{-5}$ |
| $I_l$ | 0.201 | 0.0003 |
| ξ | 2.88 | 0.01 |
| B | 0 | - |

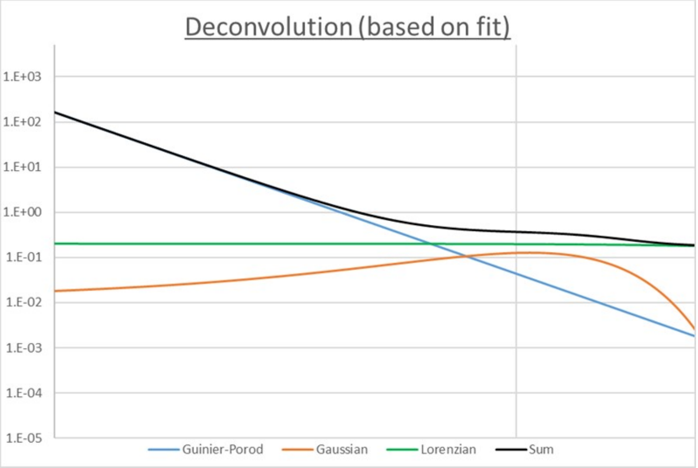

$CF_{fibre}$-$PDMAEAQ_{10\%}$@PVC

| Variable | Value | Error |
|---|---|---|
| $I_0$ | 12.7 | 16 |
| $R_g$ | 291 | 26 |
| s | 0.55 | 0.23 |
| n | 3.621 | 0.001 |
| $I_g$ | 0.1118 | 0.0003 |
| $x_0$ | 0.0494 | $6.3\ 10^{-5}$ |
| σ | 0.0213 | $7.2\ 10^{-5}$ |
| $I_l$ | 0.1794 | 0.0003 |
| ξ | 2.93 | 0.015 |
| B | 0 | - |

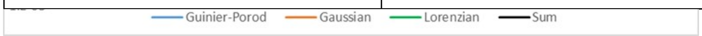


## 3. IR peaks:

The following values are the peak values obtained from the IR analysis, and their respective attributions:

Cellulose[3–5]:

| Wave number ($cm^{-1}$) | Attribution |
|---|---|
| 3340 | O-H stretching |
| 2870 - 2930 | C-H stretching |
| 1590 - 1650 | Absorbed $H_2O$ |
| 1160 | In-ring C-O-C stretching |
| 1030 | C-O stretching |

Modified Cellulose:

| Wave number ($cm^{-1}$) | Attribution |
|---|---|
| 2950 | C-H stretching |
| 2930 | |
| 2850 | |
| 2830 | |
| 1770 | |
| 1722 | C=O stretching |
| 1460 | |
| 1160 | C-N stretching |

## 4. NMR peaks:

The following values are the peak values obtained from the MNR analysis, and their respective attributions:

Cellulose:

| Chemical displacement (ppm) | Attribution |
|---|---|
| 40 | impurities |
| 56 | impurities |
| 62 | 6 |
| 65 | 6 |
| 72 | 2,3 |
| 75 | 5 |
| 84 | 4 |
| 89 | 4 |
| 101 | impurities |
| 105 | 1 |

Macro-Initiator:

| Chemical displacement (ppm) | Attribution |
|---|---|
| 26 | 9 |
| 31 | impurities |
| 41 | impurities |
| 57 | impurities |
| 62 | 6 |
| 65 | 6 |
| 72 | 2,3 |
| 75 | 5 |
| 84 | 4 |
| 89 | 4 |
| 105 | 1 |
| 172 | 7 |

CF-PDMAEA:

| Chemical displacement (ppm) | Attribution |
|---|---|
| 27 | 9 |
| 42 | 10 |
| 46 | 15 |
| 58 | impurities |
| 62 | 6,13,14 |
| 65 | 6,13,14 |
| 72 | 2,3 |
| 75 | 5 |
| 84 | 4 |
| 89 | 4 |
| 105 | 1 |
| 174 | 1,12 |

CF-DPMAEAQ:

| Chemical displacement (ppm) | Attribution |
|---|---|
| 14 | 28 |
| 23 | 27 |
| 27 | 8 |
| 31 | 17→26 |
| 33 | impurities |
| 44 | 15,10 |
| 56 | impurities |
| 60 | 6,13,14 |
| 65 | 6,13,14 |
| 72 | 2,3 |
| 75 | 5 |
| 84 | 4 |
| 89 | 4 |
| 105 | 1 |
| 175 | 7,12 |

Since the cellulose flour used contained traces of lignin, impurities were responsible for some peaks seen in NMR.

## 5. References:


1 B. Hammouda, A new Guinier–Porod model, *J Appl Crystallogr*, 2010, **43**, 716–719.

2 V. Guccini, S. Yu, M. Agthe, K. Gordeyeva, Y. Trushkina, A. Fall, C. Schütz and G. Salazar-Alvarez, Inducing nematic ordering of cellulose nanofibers using osmotic dehydration, *Nanoscale*, 2018, **10**, 23157–23163.

3 E. Singovszka, Characterization of Cellulosic Fibers by FTIR Spectroscopy for Their Further Implementation to Building Materials, *American Journal of Analytical Chemistry*, DOI:10.4236/AJAC.2018.96023.

4 C. Trilokesh and K. B. Uppuluri, Isolation and characterization of cellulose nanocrystals from jackfruit peel, *Sci Rep*, 2019, **9**, 16709.

5 O. S. Samuel, A. M. Adefusika, O. S. Samuel and A. M. Adefusika, in *Cellulose*, IntechOpen, 2019.
